# The Modern Operating Theatre

**Published:** 1910-02-26

**Authors:** 


### The Modern Operating Theatre.

The ideal hospital operating theatre, from all accounts, is yet to be designed and built, for it does not exist. This fact may seem strange to many who hold that modern advances in surgery have rendered one type of theatre indispensable; but one has only to glance at the plans of half-a-dozen of the best hospitals to realise that it is a fact. The general agreement, less than five years ago, that asepsis must be the aim of the theatre builder, was responsible for the ultra-elaboration of theatre architecture that is so marked a feature in some hospitals. It was alleged that the results obtained by operators who were rigorously aseptic, who wore gloves and goggles and ghoulish adornments, were immeasurably superior to those of operators who discarded such refinements and worked with ordinary care only. The influence of Mickulicz was, no doubt, largely responsible for this, and in all probability the old operating theatre at the Breslau surgical clinic was the prototype of the modern aseptic theatre that is now so commonly met with. The results obtained by the great German surgeon were excellent, but they could scarcely be said to have been superior to those obtained by contemporary operators who followed a less rigid routine, and while the Breslau theatre initiated the fashion in aseptic theatre building, it at the same time led surgeons seriously to consider whether or not such theatres were really necessary. At the present time there seems to be a tendency to do away with all the superfluous refinements of the strict aseptic school. In London the results obtained by surgeons working in old theatres can hardly be said to be inferior to those of operators who have at their disposal working rooms of the most pronounced aseptic type. The cost of a simple theatre of this type is much below that of the operating room required by the strict aseptic surgeon who demands that all the lights should be outside the theatre, that the warming and ventilation should be regulated by the adoption of some new and untried "aseptic" system, and that a number of structural alterations in the roof and walls should be carried out. A modern practice is to have a theatre unit which is placed quite apart from the main buildings and approached by an open corridor. It is argued that such theatres diminish the risk of bronchial troubles after the operation. This has indeed been the case at Eppendorf, where the new theatre is detached, and it would be interesting to compare the mortality statistics from this hospital with those of other institutions which still adopt the old plan.

A general comparison between all the existing operating theatres in a large city would not only be interesting in itself, but would be of decided practical value to hospital workers. There should be no great difficulty in tabulating the results of these various methods, so as to be able to judge of their relative value, and until that value is definitely and clearly apparent, there is really nothing helpful to be gleaned from a survey of the different operating theatres with a view to the designing of the ideal. At present no one can say that a particular type of theatre is unquestionably the best, and we venture to believe that if such a tabulation as we have suggested were made it would be found that the balance of favour is with types of simple design.

